# Soft-tissue grafting for peri-implantitis—a treatment option in case of unsuitable skeletal basic morphology of the alveolar bone and lack of keratinized mucosa: a retrospective clinical cohort study

**DOI:** 10.1186/s40729-015-0029-8

**Published:** 2015-10-24

**Authors:** Michael Stiller, Rainer Mengel, Sebastian Becher, Bernhard Brinkmann, Barbara Peleska, Esther Kluk

**Affiliations:** 1 School of Dental Medicine, Section of Experimental Orofacial Medicine, Philipps University, Georg-Voigt-Str. 3, 35039 Marburg, Germany; 2 School of Dental Medicine, Department of Prosthodontics, Philipps University, Georg-Voigt-Str. 3, 35039 Marburg, Germany; 3 Practice of Oral and Maxillofacial Surgery, ECDI Center, Königsallee 68, 40212 Düsseldorf, Germany; 4 Clinic ABC Bogen, ECDI Center, ABC Strasse 19, 20354 Hamburg, Germany; 5 Department of Postgraduate Education - Master of Oral Implantology, Center for Oral and Maxillofacial Surgery, Johann Wolfgang Goethe University Frankfurt, Theodor-Stern-Kai 17, Frankfurt, 60577 Germany; 6 Private Practice for Oral Surgery and Implantology, ECDI Center, Brahmsst. 11, 14193 Berlin, Germany

**Keywords:** Peri-implantitis treatment, Soft-tissue grafts, Apical skeletal basis, Keratinized mucosa

## Abstract

**Background:**

This retrospective study evaluated soft-tissue grafting as a surgical treatment option for peri-implantitis in case of unsuitable basic skeletal morphology of the alveolar bone and lack of keratinized mucosa.

**Methods:**

Twenty-eight patients (21 females, 7 males, at a mean age 59.4 years) were included with a total of 54 implants. All implants showed peri-implantitis and attached keratinized buccal mucosa of ≤2 mm. A surgical procedure of soft-tissue grafting (STG) was made by inserting an inlay and inlay-onlay transplant. Clinical investigations were made prior to the STG (baseline) and after 9–180 months (Ø 43 months) including the following parameters: soft-tissue biotype, skeletal basic morphology of the alveolar bone, width of the peri-implant keratinized mucosa (KM), mobility of the KM, pocket probing depth (PPD), and bleeding on probing (BOP).

**Results:**

Nearly all patients showed a thin soft-tissue biotype. The analysis of the skeletal basic morphology of the alveolar bone revealed a narrow apical base in 18 patients, middle base in 7 patients, and broad base in 3 patients. Width of the KM increased significantly (*p* < 0.01) from 0.4 ± 0,5 mm to 4.3 ± 1.5 mm after STG and PPD was significantly (*p* < 0,01) reduced from 6.3 ± 2,3 mm to 4.1 ± 1.9 mm. A significant reduction (*p* < 0.01) in BOP was recorded. All patients reported a clinical improvement of the inflammatory symptoms at follow-up.

**Conclusions:**

The results of this study showed that the STG can be applied successfully as a surgical treatment of peri-implantitis. It remains unclear whether soft-tissue biotype or the skeletal basic morphology of the alveolar bone affects the outcome of this surgical treatment.

## Background

With an increasing number of patients with implant-supported dentures, dentists are increasingly confronted with diseases of the peri-implant hard and soft tissue [1].

Ten-year survival rates of about 95 % of dental implants were reported, but a high percentage of these implants showed peri-implantitis [2, 3]. In this case, the complications around the implant are so complex that the mechanic-physical, biological, chemical, and toxicological effects are often difficult to determine.

In addition to the etiologically relevant patient-related as well dentist-related factors of peri-implantitis, immunological foreign body response to the implant material was described [4].

The surgical and non-surgical treatment techniques are usually based on the peri-implant bone defect morphology [5, 6].

Further, important anatomical factors such as the three-dimensional position of the implant and the resulting defect morphology in relation to the cephalometric basic pattern of the maxilla and the mandible remained unconsidered. The influence of the structural-biological factors with regard to the morphology of the peri-implant soft tissue remains also controversial [7].

Particularly, the quality and quantity of the peri-implant soft tissue affect significantly the dynamics of the marginal bone loss [8]. So that an adequate keratinized mucosa at the implant site leads to a reduced plaque accumulation, a reduced inflammatory mucosal infiltration, and a pro-inflammatory mediator release [9]. This was confirmed in an animal study of Benghazi et al., they showed in their study that in case of missed keratinized mucosa, bone resorption and prevalence of soft-tissue recession could be reduced through soft-tissue grafting [10].

The aim of this retrospective clinical cohort study is to evaluate the success of soft-tissue grafting (STG) as a surgical treatment of peri-implantitis in case of unsuitable skeletal basic morphology of the alveolar bone and the lack of keratinized mucosa. The null hypothesis of this study is that STG as an exclusive surgical treatment modality after initial treatment particularly in a case of thin skeletal basic morphology reduces clinical symptoms of peri-implantitis. In cases of an absence of the skeletal-related, three-dimensional defect, morphology in a relation to the implant position soft-tissue grafting seems to be the only reconstructive option. In such cases, STG allows recession of a peri-implant inflammatory process through stabilization of the peri-implant soft tissue.

## Methods

This study was conducted according to the guidelines of the ethics committee at the Johann Wolfgang Goethe University in Frankfurt/Main and strictly followed the ethical principles of the World Medical Association Declaration of Helsinki [11]. All patients were informed about the surgical procedure of this study and signed an informed consent.

### Patient population

The selection of the patients was made according to the data bank of the surgeon. All the patients were referred to a private clinic and to a department of oral surgery at the university. Patients who suffered from a peri-implantitis and were treated with STG were taken into consideration for this investigation. Patients were contacted by phone to ask for a participation in this study. From 62 patients, 28 agreed to participate in the study. The other 34 patients were either not available by phone or did not agree to participate in the study for personal reasons.

### Exclusion criteria

Patients with the following diseases were excluded from this study: untreated diabetes mellitus, pregnancy, bisphosphonate medication, current orthodontic treatment, tumors, and infectious diseases (HIV).

A total of 28 patients (21 females, 7 males, at a mean age 59.4 years) with a total of 54 implants were included. All patients were given a detailed description of the treatment procedures and were required to sign an informed consent form.

There was a total of 20 implants (13 in maxilla, 7 in mandible) in the anterior region (from canine to canine) and 34 implants (20 in maxilla, 14 in mandible) in the posterior region (up to the first premolar).

All implants suffered from peri-implantitis and showed attached keratinized buccal mucosa of ≤2 mm. The definition of peri-implantitis is according to established criteria defined when PPD (pocket probing depth) was >5 mm with or without BOP and with an annual bone loss of >0.2 mm [3]. The assessment of peri-implantitis was carried out 1 year after insertion of the superstructure in this study. The level of bone loss is according to the criteria of Albrektsson et al. [12] and the measurement of bleeding on probing according to the results of the study of Naert et al. [13].

To exclude falsification of a positive diagnosis of peri-implantitis in case of the absence of bleeding on probing in addition to excessive pocket probing depth patients’ discomfort and pain were strict incoming criteria for STG.The following implant systems were used for a treatment before beginning of this retrospective study: 3 × Biomet 3i (Biomet 3i Deutschland GmbH, Munich, Germany), 31 × Ankylos (Dentsply IH GmbH, Mannheim, Germany), 1 × Astra Tech (Dentsply IH GmbH, Mannheim, Germany), 15 × Camlog (Camlog Vertriebs GmbH, Wimsheim, Germany), 3 × IMZ (Friadent, Mannheim, Germany), and 1 × Nobel Replace select (Nobel Biocare Holding AG, Zürich, Switzerland).

Three patients have bar overdentures (all in maxilla), three patients telescoping dentures (one in the maxilla and two in the mandible), and 22 patients fixed prosthesis; 8 of them have single crown restorations.

The average time from the placement of the implants to the soft-tissue grafting was 63.2 ± 44.4 months.

### Treatment procedures

#### Non-surgical procedure

A sub-mucosal ultrasonic curettage with ultrasonic system (Cavitron Ultrasonic scaler® with plastic Scalers, Dentsply, Mannheim, Germany) was made by all patients to reduce the inflammation signs as prerequisite before STG. Furthermore, an antibacterial treatment was made with a sub-mucosal irrigation using hydrogen peroxide 3 % and a local antibiotic application of doxycycline (Ligosan®, Heraeus Kulzer, Hanau, Germany).

If BOP was observed after this non-surgical therapy and during the following 6 months, a surgical treatment in terms of a soft-tissue grafting using onlay or inlay-onlay grafts with respect to the skeletal configuration was conducted. In case of persistent bleeding or other significant peri-implant inflammative symptoms, the protocol prevents, from the ethical point of view, further peri-implant bone loss, excessive antibiotic administration, and occurrence of systemic influence of the pathologic process. To increase comparability and measure reliability, all the baseline measurements used in the statistical analysis were strictly performed after initial phase. All surgical procedures were carried out by one surgeon from 1998–2012.

### Surgical procedure

**Fig. 1:**
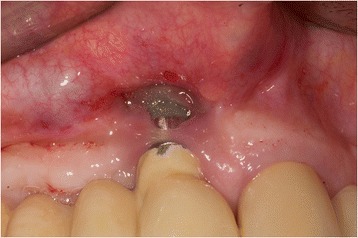
Patient 1: Buccal view of an implant regio 11. Implant (Camlog Vertriebs GmbH, Wimsheim, Germany, implant 10 years in situ) with buccal severe peri-implantitis. (8 mm PPD/pus+). Implant is partly exposed and its position is at the apical base. Reconstruction using bone grafting is therefore contraindicated. Patient (75 years old) refused explanation

The patient (Fig. 1) was anesthetized using (Ultracain® DS-forte, 1: 100,000, Sanofi-Aventis®, Frankfurt, Germany); then, a vestibular mucosal flap was raised with a preservation of a thin soft-tissue layer on the implant surface.

The thickness of the soft-tissue layer that remained on the implant surface was reduced as much as possible without perforating this layer or exposing the implant surface; for this procedure, microsurgical techniques were used in order to achieve immobility of the transplanted keratinized mucosa.

**Fig. 2:**
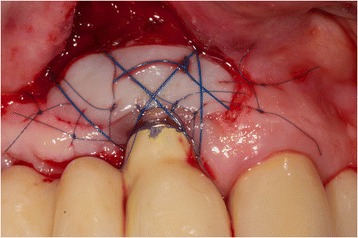
Intra-operative view after vestibulum-plasty and positioning of an onlay-transplant harvested from the palate. The coronal part of the graft was carefully fixed on the originally existing soft-tissue bridge, the apical part sits on the tissue that is denudated by vestibulum-plasty. Part of the exposed implant thread was covered directly by the graft. The graft was extended 5 mm mesially and 6 mm distally due to nutritive considerations, and it was fixed in the receptive site using suturing

**Fig. 3:**
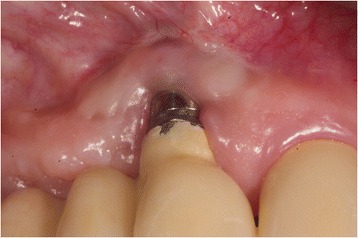
Implant 24 months after soft-tissue grafting. There is no sign of inflammation and a new mobile keratinized mucosa was built buccally, (4 mm PPD, Pu-/BOP-). Patient is symptom-free

After decontamination of the exposed implant surfaces with 37 % phosphoric acid gel (Orbis Handels-GmbH, Münster, Germany), an onlay graft was inserted (Figs. 2 and 3). In cases with highly esthetic sensitive areas, an inlay-onlay graft was used, which had, in addition to the keratinized Onlay part, a sub-epithelial connective tissue part to about 50 % [14–16]. In contrast to inlay-onlay graft, an onlay graft consists only of keratinized mucosa with a thickness of approximately 1 mm harvested from palate as well.

The grafts were harvested from the palate in the region between the first premolars and second molars. The desire extension and shape of the graft was transferred with the help of a simple, tailored paper template before graft preparation from the palate, so that it fits exactly into the previously prepared peri-implant defect and thus accurately simulated the needed amount of keratinized mucosa. The appropriate connective tissue grafts were harvested by placing the template on the palate. The grafts’ sizes were of 5–7 mm width and 7–20 mm length.

The graft was degreased with a micro scissor, and then reduced with a scalpel to about 1 mm and after that fixed on the peri-implant lesion in a stable position using non-absorbable size 5–0 and 6–0 mono sutures.

**Fig. 4:**
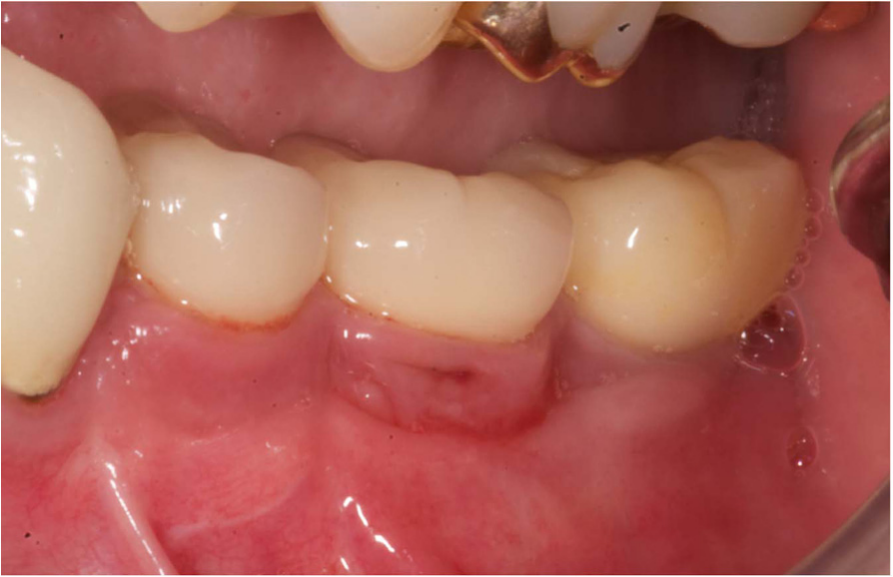
Female patient with severe peri-implantitis in regions 35, 36, 37. PPD ≥7 mm and BOP with suppuration and fistula 36, deficient keratinized mucosa due to hard tissue augmentation 5.5 years ago

**Fig. 5:**
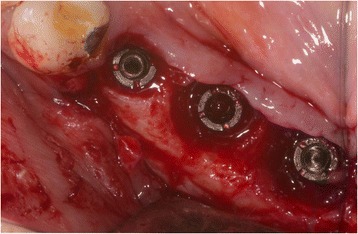
Crater-shaped peri-implant defects. Granulation tissue was removed followed by peri-implant osteotomie with the objective of optimizing subsequent soft-tissue transplantation

**Fig. 6:**
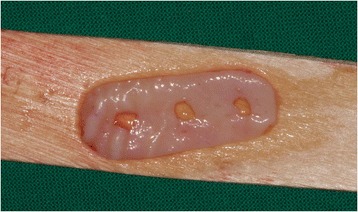
Free onlay graft harvested from the palate with perforations for sulcus formers

**Fig. 7:**
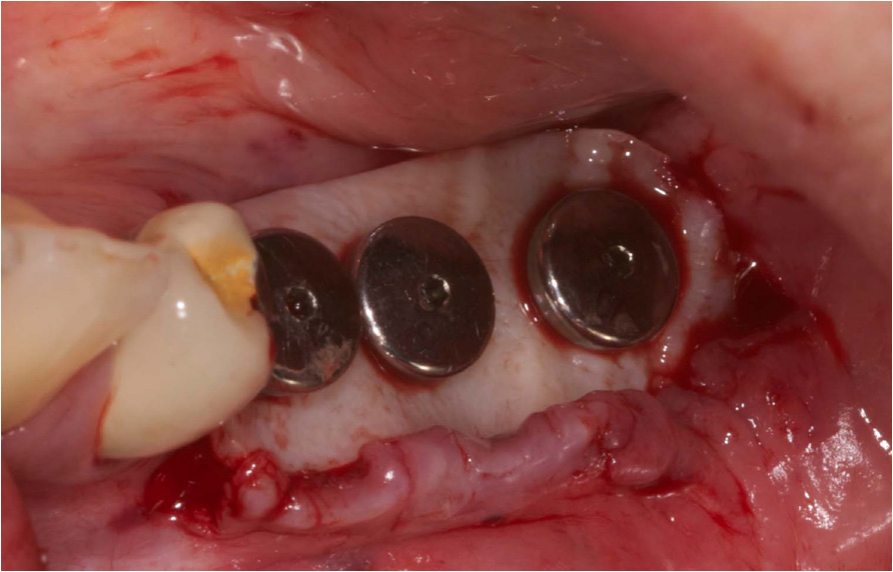
Free onlay graft try in fixed by sulcus formers after vestibular plasty at the lingual and buccal site

**Fig. 8:**
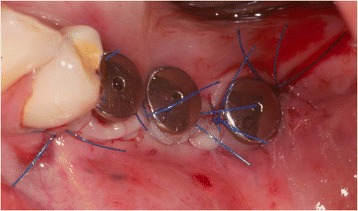
Free onlay graft buried by lingual and buccal split flap mucosa in the immediate postoperative time

**Fig. 9:**
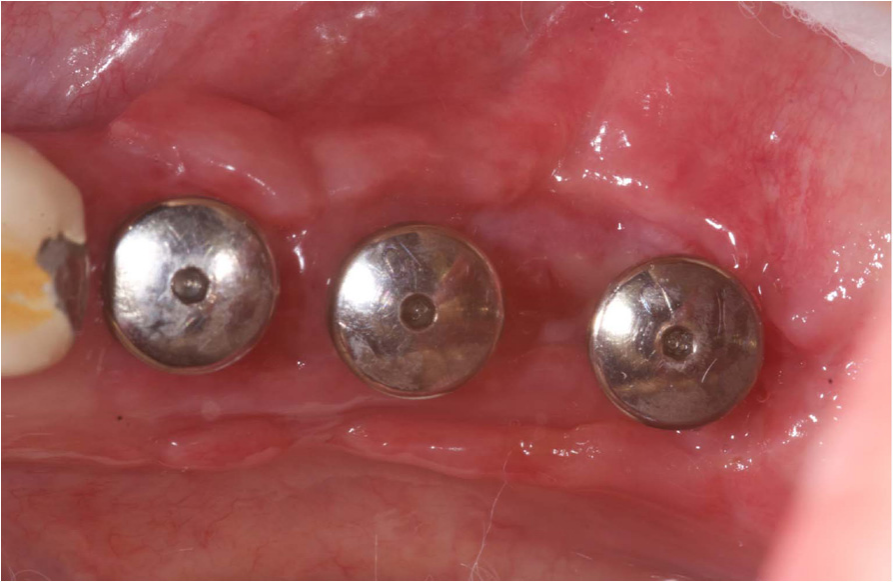
Postoperative examination after 2 weeks, perfect integration of free onlay graft with retracted wound margins at the lingual and buccal site

**Fig. 10:**
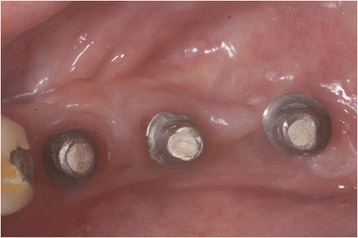
Clinical situation 2.5 years after soft-tissue transplantation without signs of inflammation

In case of crater- shaped peri-implant defects, circular soft-tissue grafts were placed improving soft-tissue quality and quantity after severe peri-implant infections with BOP and massive suppuration. Because of diminished possibility of STG fixation especially in the lower jaw, onlay grafts were buried with help of deep bucco-lingual sutures and healing abutments in the immediate postoperative period until suture removal. (Figs. 4, 5, 6, 7, 8, 9, and 10)

The palatal donor site was covered with a preoperative custom-made (0.5 mm) plastic surgical bandage.

In all patients, a postoperatively systematic antibiotic medication 3000 mg per day (1000 mg Amoxicillin Ratiopharm, Ratiopharm GmbH, Ulm, Germany) was administrated for 7 days.

To reduce postoperative wound swelling and accompanying postoperative pain, a glucocorticoid was infiltrated sub-mucosal at the vestibular region (Dexabene® 4 mg/ml, Merckle Recordati-KGaA, Darmstadt, Germany).

As analgesic treatment, all patients were given ibuprofen (IBU-ratiopharm 400 acute, Ratiopharm GmbH, Ulm, Germany). The surgical bandage and the suturing were removed after 7 to 10 days.

### Clinical parameters

The first clinical examination was carried out after initial anti-infective treatment before surgery (baseline), and the second one was made after a period of 9–180 months (Ø 43 months). In each clinical examination, the following parameters were assessed: the soft-tissue biotype, the skeletal biotype was classified, width and mobility of keratinized mucosa (KM), pocket probing depth (PPD), and bleeding on probing (BOP) as well.

The soft-tissue biotype was determined prior to the STG [17]. Differences were made between thick biotype and thin biotype. During this investigation, the transparency of the mucosa specified the suitable category when the periodontal probe was inserted into the sulcus. The probe was inserted vestibularly into the midpoint of the sulcus of an existing adjacent tooth (preferably at the maxillary central incisor), and by translucency, it was characterized as a thin biotype. A thick biotype was characterized if color impermeability through the mucosa was recognized.

Furthermore, the basic skeletal morphology of the alveolar bone was determined based on the classification of Cawood and Howell [18]. This was manually detected at the mandibular symphysis with a dental caliper (Caliper according to Beerendonk®, DCV instruments Seitingen-Oberflacht, Germany).

**Fig. 11:**
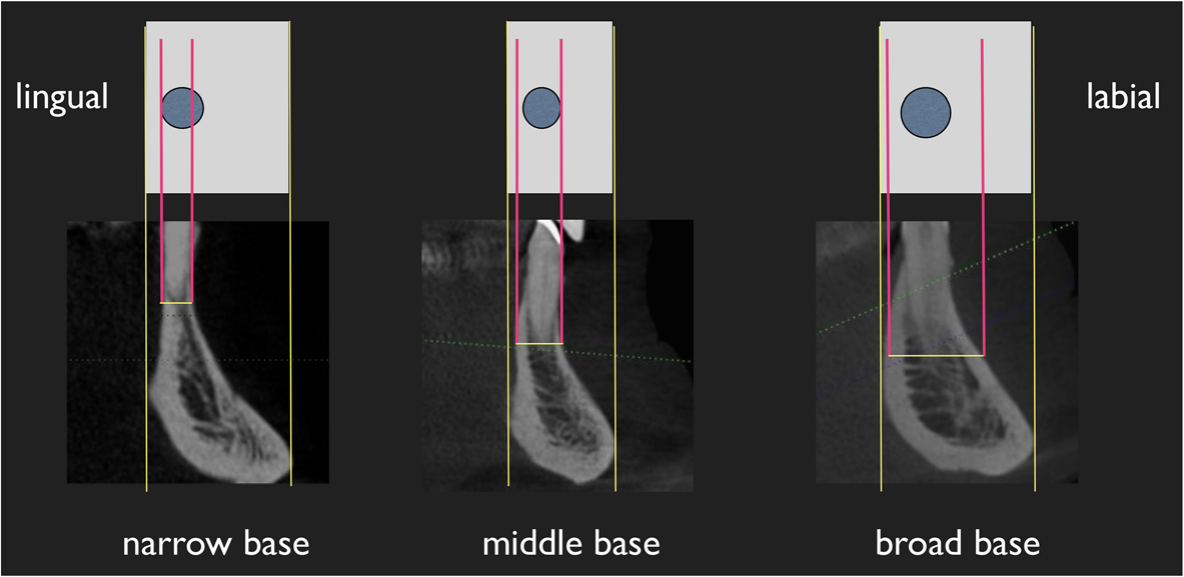
Schematic illustration of the apical base in the mandibular symphysis. A semi-quantitative measurement was made in this area. The *vertical red lines* represent the width of the alveolar bone in the apical region of the tooth/implant

A classification as a broad apical base was made when the thickness of the jaw at the apical region in oral-vestibular direction was bigger than the thickness of the jaw at the marginal area of a natural tooth. A middle base was classified, when the thickness of the jaw had the same dimension at the two regions mentioned above and when the jaw at the apical region was thinner than it was at the marginal region of a natural tooth, then it was classified as narrow. If the mandibular symphysis was edentulous, the detection of skeletal biotype was performed at the maxillary anterior region. A schematic overview of various exemplary forms of the apical bases in the symphysis region is shown in Fig. 11.

After staining of the soft tissue using the Schiller iodine solution [19], the width of the KM at each implant was measured using a periodontal probe at midpoint of the vestibular surface.

The mobility of the KM was detected using the long side of the periodontal probe by vertical movement with slight pressure. According to the mobility of the mucosa, it was classified as “movable” or as “immovable”. The worst value per implant and transplant was recorded, and in cases of more than one implant per patient, the worst value was recorded.

The PPD measurements were performed using a periodontal probe (PCP10, Hu-Friedy®, Rotterdam, Netherlands) with 25 N/mm force application on the four surfaces (mesial, distal, buccal/vestibular, and palatal/lingual). To ensure the evaluation in cases of unclear PPD values, the superstructures were removed, and PPD was measured. In patients with more than one implant, the worst PPD value was recorded.

The BOP measurements were made on four surfaces (mesial, distal, buccal/vestibular, and lingual/palatal) using the modified sulcus bleeding index by Lange et al. [20].

In addition, the suppuration was detected. The worst value per implant was recorded, and in cases of more than one implant per patient, the worst value was noted.

### Follow-up

Nine to one-hundred eighty months after STG, the following clinical parameters were assessed again: width and mobility of keratinized mucosa (KM), pocket probing depth (PPD), bleeding on probing (BOP), and suppuration (pos./neg.). All the patients enrolled in a regular maintenance program during the follow-up period.

### Statistical analysis

Collected data were documented anonymously using the Excel program (Microsoft^©^) and assessed using the statistical program SPSS 16.0 (SPSS Inc., Chicago, Illinois, USA).

For group comparisons and paired comparisons, paired *t* tests were applied with an error probability limit of 0.05. A linear regression analysis was performed as well.

## Results

All 28 patients were examined at baseline and after a period of time between 9 to 180 months. No implant was lost during this study. The soft-tissue biotype was thin in 26 of 28 patients and 2 patients as thick categorized. The analysis of the skeletal base morphology showed that 18 patients had a narrow base, 7 patients had a middle base, and 3 had a broad apical base.

**Fig. 12:**
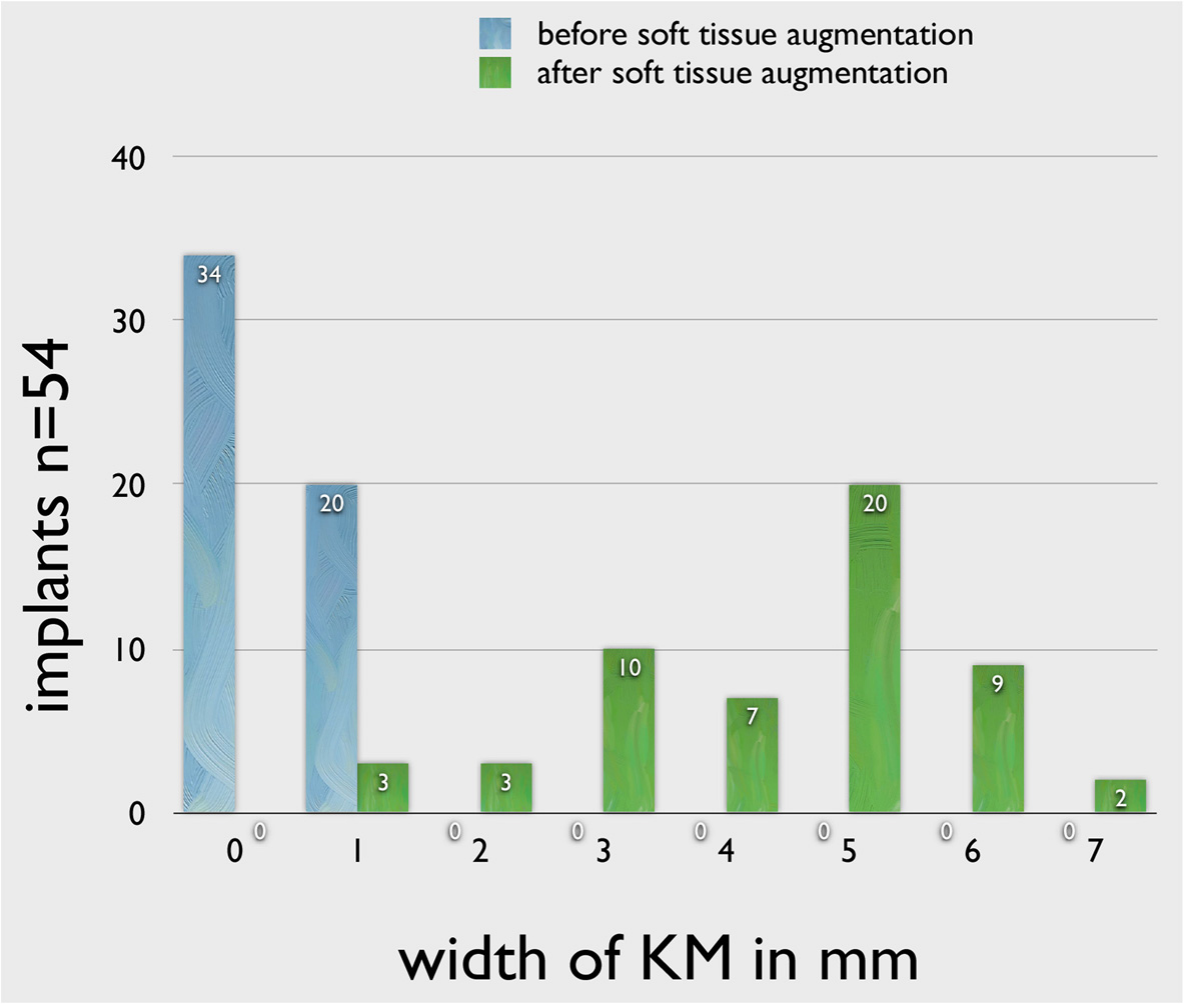
The width of the KM before and after STG. *t* test showed significant increase in KM width

The mean width of the KM was 0.4 ± 0.5 mm per implant before STG, and it was significantly (*p* < 0.01) improved to 4.3 ± 1.5 mm. Prior to the STG, 34 of 54 implants showed no keratinized mucosa, and 20 implants showed keratinized mucosa ≤1 mm. At the second measurement (follow-up), 16 implants showed KM of ≤3 mm and 38 implants showed 4–7 mm KM (Fig. 12).

High mobility of the keratinized mucosa was documented in all implants before STG. At the follow-up, 16 implants had no mobility of the KM, and 12 implants showed slight mobility.

**Fig. 13:**
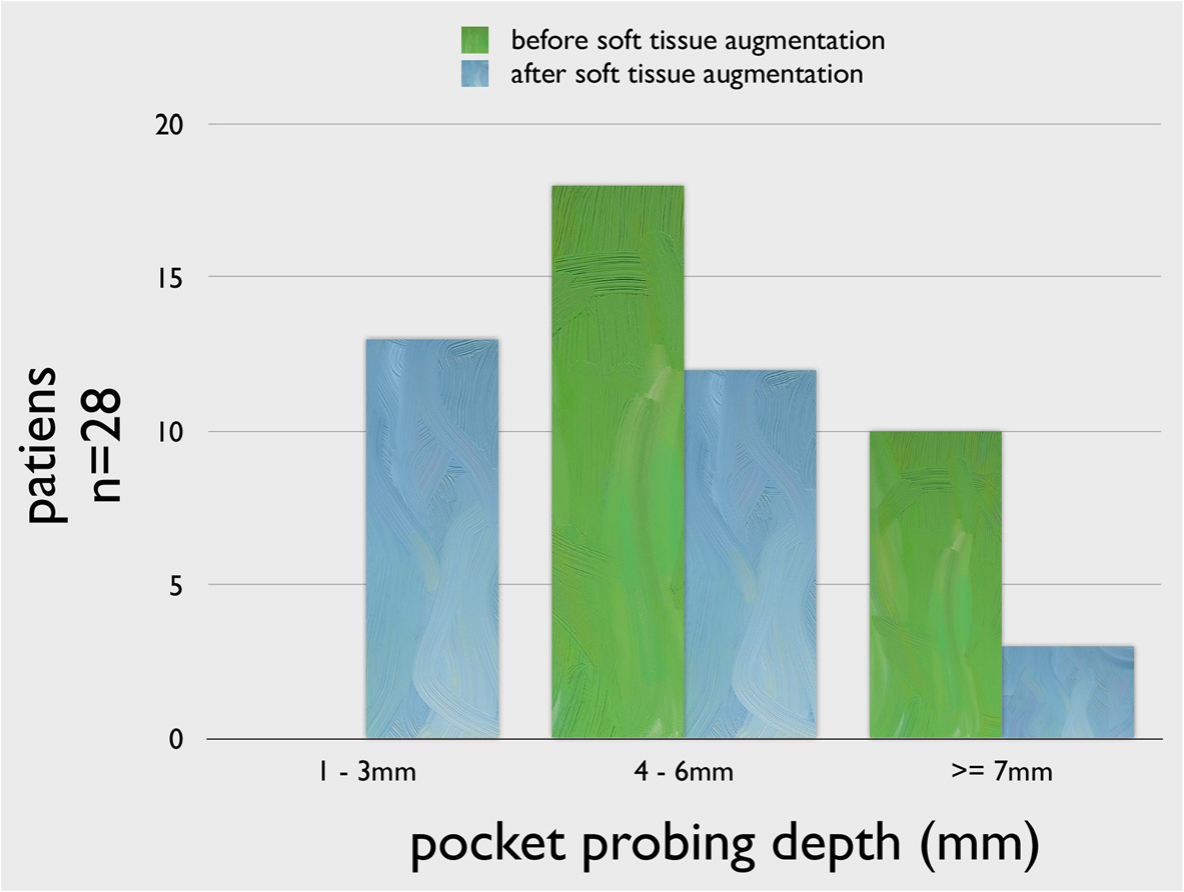
Results of the PPD before and after STG showing a significant reduction in PPD measurements (*p* < 0.01)

The mean PPD before soft-tissue augmentation was 6.3 ± 2.3 mm, and it was improved significantly (*p* < 0.01) to 4.1 ± 1.9 mm (Fig. 13). Prior to STG, 35 implants showed PPD of 4–6 mm, and 19 implants had PPD of ≥7 mm. To the time of the follow-up, the PPD was ≤3 mm at 19 implants, 4–6 mm at 30 implants, and ≥7 mm at 5 implants.

**Fig. 14:**
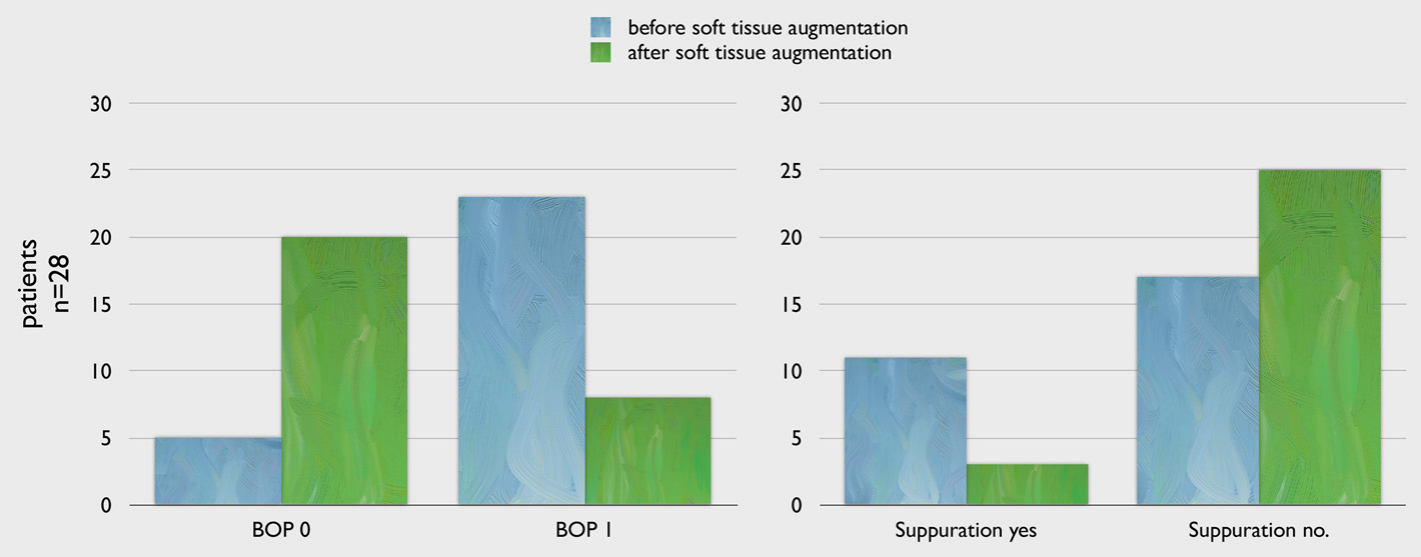
Results of BOP and suppuration before and after STG showing a significant reduction (*p* < 0.01)

At baseline, suppuration could be detected in 11 patients but at follow-up only in 3 patients (Fig. 14).

The BOP was significantly (*p* < 0.01) reduced after grafting, it was observed in 23 implants at baseline but at the follow-up only in 8 implants (Fig. 14).

Due to the wide range of the follow-up period (Ø 43 months), we underwent a stratification of the patients cohort. They were subdivided into three time intervals. First group: 6–12 month follow-up (*n* = 7), second group 13–36 month follow-up (*n* = 10), and third group >36 month follow-up (*n* = 11).

The distribution of patients was as follows: in the first group, four patients = none BOP, one patient = moderate BOP, two patients = BOP with suppuration; in the second group, eight patients = none BOP, two patients = moderate BOP; in the third group, eight patients = none BOP, two patients = moderate BOP, one patient = BOP with suppuration.

At baseline, eight patients complained about significant discomfort and six patients about slight pressure pain or burning when the area of the lesion was touched.

At follow-up, all patients were pain-free and declared a subjective stable feeling at the implant region. The positive feeling after therapy was described as follows: “stable mucosa”, “less sensitive to pain”, “absence of the food niche”, “cleaning is easier and”, “no more taste of pus”.

## Discussion

Until now, no methodology has been established as a gold standard approach for the treatment of peri-implantitis. The surgical phase comprises flap surgery or utilizing either resective or regenerative techniques. However, the selection criteria for the application of these different methods are not clearly defined.

The flap surgery with degranulation of the bony defect, decontamination of the implant surface, and postoperative antibiotic therapy could not achieve long-term stability [21]. In resective therapies, such as modeling osteoplasty, the success of the treatment depends on both the initial defect depth [22] and beginning of the disease [23]. Furthermore, the implantoplasty has been applied to smooth the supra-crestal implant surfaces [24].

The regenerative therapies aim to achieve re-osseointegration of implants, and it can be used with different materials. Long-term results are still unavailable for both of the resective and the regenerative methods, and therefore, it is still open as to which method is preferable. From a clinical point of view, these results are frustrating, and so they lead to the search for other surgical techniques, which can be used in combination with the above-mentioned methods in a certain decision-making process.

In the literature, there are no similar studies to compare with the present study. The results of the measured clinical parameters prove clearly that STGs can be applied for treatment of peri-implantitis particularly in cases of unfavorable bone defect morphology. This is the situation when the position of the planned implant is inaccurate in or outside the skeletal base of the alveolar bone. The soft-tissue grafting procedure seems to be reasonable from a palliative point of view because the peri-implant recurrent inflammatory symptoms lead to explantation with superstructure loss which affects the quality of life for patients.

Besides the soft-tissue biotype, the basic skeletal morphology of alveolar bone could be, therefore, a risk factor for the development of peri-implant disease and could also be a key factor for the successful treatment of peri-implantitis using soft-tissue grafting. The fact, that in the present cohort study approximately 65 % of patients have narrow apical base and only 10 % showed a broad base, lead to the suggestion that a narrow skeletal configuration of the upper and lower jaw as class IV according to Cawood and Howell [18] can be seen as an additional possible risk factor for the development of peri-implant inflammatory lesions. The reduced bone dimension above ossointegrated implants with respect to buccal and lingual site in case of narrowing with reduced bone vascularization especially in case of dense bone could be responsible for progressive bone loss and superinfection in such unfavorable skeletal configuration. In the literature, there are no references confirming that with certain skeletal conditions higher incidence rates can be expected. It is therefore advisable that future clinical trials consider this feature as a clinical evaluation criterion to validate this observation with clinical data. In recent years and especially in orthodontics, it is actively discussed that the development of the alveolar bone at different basic skeletal constitutions has a significant relation to the application of forces. It was found that fenestrations and dehiscences at orthodontically moved teeth will mainly be determined by the skeletal morphology, the corresponding growth pattern, and the bite situation [25, 26]. For example, patients with a horizontal skull structure and a broad apical alveolar bone base showed quantitatively less fenestrations and dehiscences of teeth than patients with a vertical skull structure and concomitant thin apical bone base. The importance of the position of the implant in relation to the skeletal basic morphology of the alveolar bone as a risk factor is still not clear. Further risk analysis using the predictive value of anatomical factors in relation to cephalometric basic pattern of the maxilla and mandible in the development of the peri-implantitis is advisable.

Similar to the fact that peri-implant lesions are quite different from periodontitis is the reaction behavior of the alveolar bone adjacent to the teeth completely different from its behavior at the implant sites. Although, it can be accepted that fenestrations and dehiscences occur at implants in the form of severe peri-implant inflammatory defects when the implants are set at or even outside the bone base. In this case, hardly reconstructable peri-implant defect morphology will result due to the 3D missed bone.

In spite of the fact that the quality and quantity of the peri-implant soft tissue has a very important effect on the marginal peri-implant bone loss [10, 27–30], there are no approaches in the literature for the soft-tissue grafting as a therapeutic method which can be used alone after initial treatment. The present study showed that in most cases and even by undesired peri-implant defect morphologies, stabilization of the soft tissue and reduction of the peri-implant inflammatory process could be achieved.

The reduced mobility of the soft tissue achieved in this study allows the conclusion that the presented methodology is strongly technical and surgeon sensitive. The degree of preoperative inflammatory activity did not correlate with the degree of immobilization of the KM gained, which means that the degree of postoperative gained immobility was not dependent on the severity of preoperative bleeding. The high proportion of patients with a thin mucosal biotype and the achieved changes in the local peri-implant biotypes emphasize this observation. In this context, the consequent anti-infective treatment performed before STG is of prime importance for patient outcome. Reduction of local bleeding and vulnerability allows proper transplant procedure and a reliable healing process.

It remains unsettled whether patients with a thick biotype will be less responsive to soft-tissue grafting than patients with a thin biotype. Therefore, it is recommended to conduct such investigation with a control group of patients with thick biotype in order to be able to answer this question. It is also recommended to investigate other approaches in larger cohort constitutional variations with different phenotypic forms of intra-oral soft and hard tissues of different ethnic groups similar to the study by Patil et al. [31].

According to the follow-up data that were stratified, it revealed that in the shorter period of follow-up, BOP could be rather present than in the longer healing period (>12 month, 2nd and 3rd group). The reason for that could be the slow-going scarification of the transplanted mucosa for many years. Depending on the increasing rigidity of the transplanted keratinized mucosa, the irritation of the mobile mucosa decreases and the signs of inflammation (BOP) is reduced. Besides the weak data, it seems that the new mucosa has to undergo a maturing process before a significant reduction of inflammatory symptoms occurs. Nevertheless, the study design cannot clearly answer this question; further controlled prospective trials have to be performed.

The reduction of PPD was possible through the stabilization and immobilization of the soft tissue accompanied by the establishment of an inflammation-free soft-tissue cuff, more than through the bone regeneration. The significant reduced values of BOP values are in accordance with the results of Boynueğri et al. [9] which confirmed that an adequate width of keratinized mucosa reduced plaque accumulation, inflammatory mucosal infiltration, and pro-inflammatory mediator release. Whether the STG at dental implants finally influences crestal bone changes remains unclear until now. Further controlled clinical trials with regular x-ray evaluation would be of tremendous interest.

## Conclusions

Considering the results of this retrospective study, it can be summarized that soft-tissue grafting as an additional surgical therapy option can be integrated in the treatment concept of peri-implantitis. The results showed as well that with certain clinical morphological conditions, the soft-tissue grafting can enhance a successful surgical treatment of peri-implantitis. It can be accepted as well that the implant system, and thus, the implant design has no direct influence on the indication of STG.

The soft-tissue grafting procedure seems to be reasonable from a palliative point of view because the peri-implant recurrent inflammatory symptoms lead to explantation with superstructure loss which affect the quality of life for patients.

## Abbreviations

BOP: bleeding on probing
KM: keratinized mucosa
PPD: pocket probing depth
STG: soft-tissue grafting

## References

[1] Atieh MA, Alsabeeha NH, Faggion CM, Duncan WJ (2013). The frequency of peri-implant diseases: a systematic review and meta-analysis. J Periodontol.

[2] Mombelli A, Müller N, Cionca N (2012). The epidemiology of peri-implantitis. Clin Oral Implants Res.

[3] Swierkot K, Lottholz P, Flores-de-Jacoby L, Mengel R (2012). Mucositis, peri-implantitis, implant success, and survival of implants in patients with treated generalized aggressive periodontitis: 3- to 16-year results of a prospective long-term cohort study. J Periodontol..

[4] Albrektsson T, Buser D, Chen ST, Cochran D, DeBruyn H, Jemt T, Koka S, Nevins M, Sennerby L, Simion M, Taylor TD, Wennerberg A (2012). Statements from the Estepona Consensus Meeting on Peri-implantitis, 2012. Clin Implant Dent Relat Res.

[5] Khoshkam V, Chan HL, Lin GH, MacEachern MP, Monje A, Suarez F, Giannobile WV, Wang HL (2013). Reconstructive procedures for treating peri-implantitis: a systematic review. J Dent Res.

[6] Lang NP, Wilson TG, Corbet EF (2000). Biological complications with dental implants: their prevention, diagnosis and treatment. Clin Oral Implants Res..

[7] Greenstein G, Cavallaro J (2011). Failed dental implants: diagnosis, removal and survival of reimplantations. J Am Dent Assoc.

[8] Lin GH, Chan HL, Wang HL (2013). The significance of keratinized mucosa on implant health: a systematic review. J Periodontol.

[9] Boynueğri D, Nemli SK, Kasko YA (2013). Significance of keratinized mucosa around dental implants: a prospective comparative study. Clin Oral Implants Res.

[10] Bengazi F, Botticelli D, Favero V, Perini A, Urbizo Velez J, Lang NP (2014). Influence of presence or absence of keratinized mucosa on the alveolar bony crest level as it relates to different buccal marginal bone thicknesses. An experimental study in dogs. Clin Oral Implants Res.

[11] World Medical Association (2013). World Medical Association Declaration of Helsinki: ethical principles for medical research involving human subjects. JAMA..

[12] Albrektsson T, Zarb G, Worthington P, Eriksson AR (1986). The long-term efficacy of currently used dental implants: a review and proposed criteria of success. Int J Oral Maxillofac Implants.

[13] Naert I, Gizani S, Vuylsteke M, van Steenberghe D (1998). A 5-year randomized clinical trial on the influence of splinted and unsplinted oral implants in the mandibular overdenture therapy. Part I: Peri-implant outcome. Clin Oral Implants Res.

[14] Seibert JS, Louis JV (1996). Soft tissue ridge augmentation utilizing a combination onlay-interpositional graft procedure: a case report. Int J Periodontics Restorative Dent.

[15] Stiller M, Eisenmann E, Fritz H, Freesmeyer WB (1998). Der lokale Alveolarkammaufbau bei Weichgewebsdefiziten. Z Zahnärztl Implantol..

[16] Stiller M (2001). Die Rekonstruktion des mukogingivalen Komplexes an Einzelzahnimplantaten zur Verbesserung von Funktion und Ästhetik mit Hilfe mikrochirurgischer Wundversorgungstechniken. ZWR - Das deutsche Zahnärzteblatt..

[17] De Rouck T, Eghbali R, Collys K, De Bruyn H, Cosyn J (2009). The gingival biotype revisited: transparency of the periodontal probe through the gingival margin as a method to discriminate thin from thick gingiva. J Clin Periodontol..

[18] Cawood JI, Howell RA (1988). A classification of the edentulous jaws. Int J Oral Maxillofac Surg.

[19] Kern G, Stadler G, Hinderfeld E (1962). Die Schillersche Jodprobe. Archiv für Gynäkologie.

[20] Lange DE, Plagmann HC, Eenboom A, Promesberger A (1977). Clinical methods for the objective evaluation of oral hygiene. Dtsch Zahnarztl T.

[21] Heitz-Mayfield LJ, Lang NP (2010). Comparative biology of chronic and aggressive periodontitis vs. peri-implantitis. Periodontol 2000.

[22] Serino G, Turri A (2011). Outcome of surgical treatment of peri-implantitis: results from a 2-year prospective clinical study in humans. Clin Oral Implants Res.

[23] Charalampakis G, Rabe P, Leonhardt A, Dahlén G (2011). A follow-up study of peri-implantitis cases after treatment. J Clin Periodontol.

[24] Schwarz F, John G, Mainusch S, Sahm N, Becker J (2012). Combined surgical therapy of peri-implantitis evaluating two methods of surface debridement and decontamination. A two-year clinical follow up report. J Clin Periodontol.

[25] Enhos S, Uysal T, Yagci A, Veli İ, Ucar FI, Ozer T (2012). Dehiscence and fenestration in patients with different vertical growth patterns assessed with cone-beam computed tomography. Angle Orthod.

[26] Evangelista K, Vasconcelos Kde F, Bumann A, Hirsch E, Nitka M, Silva MA (2010). Dehiscence and fenestration in patients with Class I and Class II Division 1 malocclusion assessed with cone-beam computed tomography. Am J Orthod Dentofacial Orthop.

[27] Gobbato L, Avila-Ortiz G, Sohrabi K, Wang CW, Karimbux N (2013). The effect of keratinized mucosa width on peri-implant health: a systematic review. Int J Oral Maxillofac Implants.

[28] Linkevicius T, Apse P, Grybauskas S, Puisys A (2009). The influence of soft tissue thickness on crestal bone changes around implants: a 1-year prospective controlled clinical trial. Int J Oral Maxillofac Implants.

[29] Linkevicius T, Puisys A, Linkeviciene L, Peciuliene V, Schlee M. Crestal bone stability around implants with horizontally matching connection after soft tissue thickening: a prospective clinical trial. Clin Implant Dent Relat Res. 2013;17. doi:10.1111/cid.12155. [Epub ahead of print]. 10.1111/cid.12155 24103157

[30] Brito C, Tenenbaum HC, Wong BK, Schmitt C, Nogueira-Filho G (2014). Is keratinized mucosa indispensable to maintain peri-implant health? A systematic review of the literature. J Biomed Mater Res B Appl Biomater.

[31] Patil R, van Brakel R, Mahesh K, de Putter C, Cune MS (2013). An exploratory study on assessment of gingival biotype and crown dimensions as predictors for implant esthetics comparing caucasian and Indian subjects. J Oral Implantol.

